# Poor reporting of physical activity and exercise interventions in youth mental health trials: A brief report

**DOI:** 10.1111/eip.13045

**Published:** 2020-09-13

**Authors:** Michaela C. Pascoe, Alan P. Bailey, Melinda Craike, Tim Carter, Rhiannon K. Patten, Nigel K. Stepto, Alexandra G. Parker

**Affiliations:** ^1^ Institute for Health and Sport Victoria University Melbourne Victoria Australia; ^2^ Peter MacCallum Cancer Centre Melbourne Victoria Australia; ^3^ Orygen, and Centre for Youth Mental Health The University of Melbourne Parkville Victoria Australia; ^4^ Mitchell Institute Victoria University Melbourne Victoria Australia; ^5^ Institute of Mental Health, School of Health Sciences University of Nottingham Nottingham UK

**Keywords:** exercise, mental health, physical activity, reporting standards, youth

## Abstract

**Aim:**

To describe the quality and completeness of the description and reporting of physical activity and exercise interventions delivered to young people to promote mental health or treat mental illness.

**Methods:**

We conducted a series of scoping reviews identifying 64 controlled trials of physical activity and exercise interventions delivered to young people. We extracted: intervention characteristics, personnel and delivery format, the intensity, duration, frequency and type of physical activity or exercise.

**Results:**

There was limited reporting of intervention details across studies; 52% did not provide information to confidently assess intervention intensity, 29% did not state who delivered the intervention, and 44% did not specify the intervention delivery format.

**Conclusions:**

We recommend that authors adhere to the CONSORT reporting requirements and its intervention reporting extensions, (a) the Template for Intervention Description and Replication, (b) Consensus for Exercise Reporting Template and (c) as part of this, detail the frequency, intensity, time and type of physical activity recommendations and prescriptions. Without this, future trials are unable to replicate and extend previous work to support or disconfirm existing knowledge, leading to research waste and diminishing translation and implementation potential.

## 1 INTRODUCTION

Youth mental disorders are a leading cause of morbidity and mortality in young people (Mokdad et al., 2016). Therefore, the promotion of mental health and the prevention of mental health disorders are paramount. Young people are often reluctant to seek help for mental health concerns, therefore interventions need to be youth‐friendly, acceptable, feasible, non‐stigmatizing (Rickwood, Deane, Wilson, & Ciarrochi, 2005) and matched to their specific needs (McGorry, Goldstone, Parker, Rickwood, & Hickie, 2014). Access to effective interventions that are youth appropriate have the potential to rapidly improve the mental health and functioning of young people (McGorry et al., 2014). Physical activity/exercise is a non‐stigmatizing intervention with few side‐effects (Chu, Buckworth, Kirby, & Emery, 2009) and is viewed by young people as helpful in promoting mental health and treating mental health problems (Jorm & Wright, 2007). We recently conducted a series of scoping reviews (M. Pascoe et al., 2020; M. C. Pascoe et al., 2020a; M. C. Pascoe et al., Under Review) to examine the evidence for physical activity/exercise as a mental health promotion strategy and treatment approach for mental health disorders in young people (mean age 12‐25.9 years). As part of this work, we extracted data from each included study for physical activity/exercise intensity and delivery format. Through this, we found a general paucity of clear specification and reporting of physical activity interventions for mental health promotion and treatment. The characteristics of descriptions and reporting are presented and discussed in the current brief report.

## 2 METHODS

We conducted a series of three scoping reviews (M. Pascoe et al., 2020; M. C. Pascoe et al., 2020a; M. C. Pascoe et al., Under Review) in line with PRISMA‐ScR guidelines (Tricco et al., 2018) and following the five stage framework outlined by Arksey and O'Malley (2005). To determine study eligibility, the following criteria were applied to the studies identified in the initial search: population mean age between 12 and 25.9 years; published from 1980 to 2017, included a physical activity or exercise intervention and a comparison or control condition; reported at least one mental health symptom outcome, such as depression symptoms (American College of Sports Medicine, 1998; Norton, Norton, & Sadgrove, 2010); study designs were randomized or non‐randomized controlled trials and studies were published in English. Excluded studies were unpublished studies and non‐intervention studies.

### 2.1 Information sources

We conducted searches using ‘Evidence Finder’ (www.orygen.org.au), which is a comprehensive database of all available published controlled trials and systematic reviews of interventions in the youth mental health field (De Silva, Bailey, Parker, Montague, & Hetrick, 2018; Hetrick, Parker, Callahan, & Purcell, 2010). The searchable database is populated annually using comprehensive and systematic searches of the Embase, MEDLINE, PsycINFO and Cochrane Library databases, coupled with strict and reproducible inclusion criteria to identify studies. It includes research published from 1980 to 2017 and contains all available prevention, treatment and relapse‐prevention studies in young people (mean age 6‐25 years), across the following mental illnesses: anxiety, depression, bipolar, eating disorders, psychosis, substance use and suicide‐self harm. It contains controlled trials (including randomized controlled trials and quasi‐randomized studies), systematic reviews and meta‐analyses, published in English. Unpublished trials are not included within the Evidence Finder. The following criteria were applied to the search engine (https://www.orygen.org.au/Training/Evidence-Finder): (a) mental health or substance use problem: ‘all’; (b) stage of illness: ‘all’; (c) treatment/intervention: ‘complementary and alternative interventions’, followed by ‘Physical activity/exercise’; (d) publication date: ‘all’. Title/abstract and then full text screening were independently undertaken by at least two authors. Data charting (Arksey & O'Malley, 2005) was undertaken by a single author using a specifically designed extraction form and was checked by a second author. Reference lists of identified literature were searched for suitable primary research based on titles in the first instance, and if relevant abstracts and full text review.

Title/abstract screening was undertaken by two authors (Michaela C. Pascoe and Alan P. Bailey). Full texts were independently reviewed by two authors (Michaela C. Pascoe, Alan P. Bailey and Melinda Craike). There were no conflicts. Data charting (Arksey & O'Malley, 2005) was undertaken by a single author (Michaela C. Pascoe) using a specifically designed extraction form (Table 1) and extraction was checked by a second author (Tim Carter and Alan P. Bailey). Data were obtained directly and only from the published articles.

**TABLE 1:** Reporting of the characteristics of the physical activity interventions

Study	Was the physical activity or exercise intensity reported?	Was the physical activity or exercise type (ie, aerobic/resistance and/or form, ie, yoga, taichi) adequately reported?	Was the personnel delivering intervention reported?	Was it stated if the intervention was delivered in an Individual or group format?	Was dosage (both duration and frequency) reported?
Aras and Ewert (2016)	✕	✓	✓	✕	✓
Baghurst and Kelley (2014)	✕	✕	✓	✓	✓
Balchin, Linde, Blackhurst, Rauch, and Schonbachler (2016)	✓	✓	✕	✕	✓
Balkin, Tietjen‐Smith, Caldwell, and Shen (2007)	✕	✓	✕	✕	✕
Bao and Jin (2015)	✕	✓	✓	✓	✓
Bartholomew (1999)	✓	✓	✓	✓	✓
Berger, Friedmann, and Eaton (1988)	✓	✓	✕	✓	✓
Bonhauser et al. (2005)	✕	✕	✓	✓	✓
Broman‐Fulks, Berman, Rabian, and Webster (2004)	✓	✓	✓	✓	✓
Broman‐Fulks and Storey (2008)	✓	✓	✓	✕	✓
Brown, Welsh, Labbe, Vitulli, and Kulkarni (1992)	✕	✕	✕	✕	Duration ns
Carei, Fyfe‐Johnson, Breuner, and Brown (2010)	✕	✓	✓	✓	✓
Carter et al. (2015)	✓	✓	✓	✓	✓
Cecchini‐Estrada, Mendez‐Gimenez, Cecchini, Moulton, and Rodriguez (2015)	✕	✕	✓	✓	✓
Curtis et al. (2016)	✓	✓	✓	✓	✕
Daley, Copeland, Wright, Roalfe, and Wales (2006)	✓	✓	✓	✓	✓
del Valle et al. (2010)	✕	✓	✓	✓	✓
Fallon and Hausenblas (2005)	✓	✓	✕	✓	✓
Focht, Koltyn, and Bouchard (2000)	✓	✓	✕	✕	✓
Gallego, Aguilar‐Parra, Cangas, Langer, and Manas (2015)	✕	✕	✓	✕	✓
Hemat‐Far, Shahsavari, and Mousavi (2012)	✓	✓	✓	✕	✓
M. P. Herring, Jacob, Suveg, Dishman, and O'Connor (2011); Matthew P. Herring, Jacob, Suveg, and O'Connor (2011)	✓	✓	✓	✕	✓
Hilyer et al. (1982)	✕	✓	✓	✓	✓
Hopkins (2012)	✕	✓	✕	✕	✓
Hughes et al. (2013)	✓	✓	✓	✕	✓
Jeong et al. (2005)	✕	✓	✕	✕	✓
Julian (2012)	✓	✓	✕	✕	✓
Khalsa, Hickey‐Schultz, Cohen, Steiner, and Cope (2012)	✕	✕	✓	✓	✓
Khorvash (2012)	✕	✓	✕	✕	✓
Lindheimer, O'Connor, McCully, and Dishman (2017)	✓	✓	✕	✕	✓
Loh, Abdullah, Abu Bakar, Thambu, and Nik Jaafar (2015)	✕	✓	✓	✓	✓
MacMahon and Gross (1988)	✕	✕	✓	✓	✓
Mason and Asmundson (2018)	✓	✓	✓	✕	✓
Medina et al. (2014); J. A. Smits et al. (2008)	✓	✓	✓	✓	✓
Melnyk et al. (2009)	✕	✕	✕	✓	✓
Melnyk et al. (2013)	✕	✕	✓	✓	Duration ns
Mothes et al. (2017)	✓	✓	✓	✕	✓
Nabkasorn et al. (2006)	✓	✓	✓	✓	✓
Noggle, Steiner, Minami, and Khalsa (2012)	✕	✕	✓	✓	✓
Noorbakhsh and Alijani (2013)	✕	✓	✕	✕	✓
Norris, Carroll, and Cochrane (1992)	✓	✓	✓	✓	✓
O'Dougherty, Hearst, Syed, Kurzer, and Schmitz (2012)	✓	✓	✓	✓	✓
Olson, Brush, Ehmann, and Alderman (2017)	✓	✓	✓	✕	✓
Parker et al. (2016)	✕	✕	✓	✓	✓
Robledo‐Colonia (2012)	✓	✓	✓	✓	✓
Roshan, Pourasghar, and Mohammadian (2011)	✓	✓	✕	✕	Duration ns
Roth and Holmes (1987)	✓	✓	✓	✓	✓
Roth (1989)	✓	✓	✓	✕	✓
Roth, Bachtler, and Fillingim (1990)	✕	✓	✕	✓	✓
Rotheram‐Borus et al. (2016)	✕	✓	✓	✓	Duration ns
Sabourin, Watt, Krigolson, and Stewart (2016)	✓	✓	✓	✓	✓
Sadeghi et al. (2016)	✕	✓	✓	✕	Frequency ns
Smith, Greer, Sheets, and Watson (2011)	✕	✓	✓	✓	✓
J. A. J. Smits, Meuret, Zvolensky, Rosenfield, and Seidel (2009)	✓	✓	✕	✕	✓
Sundgot‐Borgen, Rosenvinge, Bahr, and Schneider (2002)	✓	✓	✓	✓	✓
Taspinar, Aslan, Agbuga, and Taspinar (2014)	✕	✓	✓	✕	✓
Ventura et al. (2013)	✕	✕	✕	✕	✓
Weinstock, Capizzi, Weber, Pescatello, and Petry (2014)	✓	✓	✓	✕	✓
Weinstock, Petry, Pescatello, and Henderson (2016)	✕	✓	✓	✓	Frequency ns
Wipfli (2011)	✕	✓	✓	✕	✓
Woolery, Myers, Sternlieb, and Zeltzer (2004)	✕	✓	✓	✓	✓
Wunram et al. (2018)	✓	✓	✓	✓	✓
Yang, Zhai, Gao, and Zhang (2015)	✕	✕	✕	✓	✓
Yavari (2008)	✕	✓	✕	✕	Duration ns

*Notes:* In two instances, two studies reported different outcomes from the same trial in separate publications, and therefore we have combined them in our review (Medina et al., 2014; J. A. Smits et al., 2008) and (Herring, Jacob, Suveg, Dishman, and O'Connor (2011); Matthew P. Herring, Jacob, Suveg, and O'Connor (2011).

Abbreviations: ✕, not reported; ✓, reported; ns, not stated.

Two authors reviewed each study for objective (heart rates [HR], %maximal HR, %HR reserve, %1‐repetition maximum, percent of maximal‐oxygen‐uptake [%VO_2max_]) and subjective (ratings of perceived exertion) measures of exercise intensity, and classified intervention intensity for aerobic (Norton et al., 2010) and resistance exercise (Garber et al., 2011). Where interventions were poorly described, we attempted to estimate an exercise intensity based on the compendium of exercise energy expenditure (Ainsworth et al., 2000). A critical appraisal of individual sources of evidence was not conducted in the current scoping review.

## 3 RESULTS

We identified a total of 64 controlled trials that investigated physical activity as a treatment for mental illness or mental health promotion strategy among young people. As shown in Table 1, we found a general paucity of reporting of the characteristics of the physical activity interventions. We found that only 31 (48%) provided sufficient information to accurately assess the intensity of the intervention with regards to objective (HR, %maximal HR, %HR reserve, %1‐repetition maximum, %VO_2max_) and subjective (ratings of perceived exertion) measures of exercise intensity (Garber et al., 2011; Norton et al., 2010). We were unable to determine the intervention intensity in 33 (52%) of the studies. Only 51 (80%) of studies stated the physical activity or exercise type (ie, aerobic/resistance and/or form, that is, yoga, tai chi).

The delivery format was also poorly described; 19 (29%) did not state who delivered the intervention; 28 (44%) did not specify if the intervention was delivered in a group or individual format; 9 (14%) did not report the intervention dose as defined by both duration and frequency.

## 4 DISCUSSION

This report highlights an important limitation in the literature and a need for better specification and clearer reporting of physical activity interventions for mental health promotion and treatment for youth.

Many existing studies have failed to specify intervention intensity and delivery format. This has limited the interpretation and application of the evidence base for physical activity/exercise as a treatment for mental illness or mental health promotion strategy. It is clear from our scoping reviews, and previous research, that a variety of modalities and intensities may produce positive effects, and that factors such as delivery in group or individual formats may potentially explain some of the positive effects seen in physical activity/exercise interventions. However, due to poor reporting, it remains unclear if the intensity and the type of activity are important for mental health benefits. Therefore, although different intensities and different physical activity/exercise modalities may produce different effects on mental health outcomes, current poor reporting likely hinders efforts to identify which of these components, if any, are driving change. Better reporting in future studies will shed light on the role each plays on mental health outcomes and facilitate implementation in practice.

Whether the type and intensity of the physical activity engaged in is prescribed or self‐selected also should be specified. Autonomy is proposed as one of three basic psychological needs fundamental to positive mental health (Craft, Perna, Freund, & Culpepper, 2008; Ryan & Deci, 2000) and participants experience a greater psychological tolerance to higher intensity physical activity when intensity is self‐selected, rather than imposed (Ekkekakis, Parfitt, & Petruzzello, 2011). The environment in which the intervention is delivered should also be clearly stated, given that there is evidence indicating that physical activity undertaken in natural environments may have a more positive mental health benefit compared to physical activity undertaken indoors (Coon et al., 2011). Finally, it is important to report who delivered the intervention, as our scoping reviews show that this varies greatly between studies and can include exercise physiologists, personal trainers, psychologists, researchers and school teachers (M. Pascoe et al., 2020; M. C. Pascoe et al., 2020a; M. C. Pascoe et al., Under Review). The person delivering the intervention may influence intervention fidelity, the cost of delivering the intervention, and external validity, or its potential to be delivered in practice.

### 4.1 Reporting recommendations

We recommend that in addition to adhering to the minimum reporting requirements set out in the CONSORT statement (Moher et al., 2010; Schulz, Altman, Moher, & the CONSORT Group, 2010) that authors should follow its intervention reporting extensions, (a) the Template for Intervention Description and Replication (TIDieR) (T. Hoffmann et al., 2016) and (b) Consensus for Exercise Reporting Template (CERT) (Slade, Dionne, Underwood, & Buchbinder, 2016; Slade Dionne, Underwood, Buchbinder, Beck, et al., 2016). We recommend trialists adhere to these established standards. As part of this reporting, we recommend that future studies better specify their intended intervention characteristics and delivery format, ensuring clear statements that detail the frequency, intensity, time and type (FITT) principles of physical activity recommendations and prescriptions. Studies should also measure and report participant engagement in intended physical activity/exercise and dose, ensuring that FITT principles are appropriately tracked and quantified using subjective and device‐based measures (eg, perceived exertion, actigraphy, heart rate monitoring).

We also acknowledge that self‐selected and therefore variable physical activity interventions, as well as behaviour change interventions aimed to increase physical activity and exercise, may be unable to meet the recommended reporting requirements. Most of the current recommendations apply to prescribed exercise/physical activity interventions, however, self‐selected variable physical activity interventions, as well as behaviour change interventions, could still measure changes in engagement in physical activity levels and report on this as an outcome (objective and subjective measures of physical activity).

Although this guidance is not new, poor intervention reporting has important implications for the field today and into the future (Glasziou et al., 2014; Glasziou, Meats, Heneghan, & Shepperd, 2008; T. C. Hoffmann, Erueti, & Glasziou, 2013). First, poor reporting hinders the identification of core components from potentially effective interventions, while preventing the comparison of components across trials to identify ‘best bets’ for implementation (Glasziou et al., 2008). Poor reporting prevents replication and delays progress in the research field as replication of previous work is important to confirm or reject existing knowledge and is the building block to extending current knowledge by adding, subtracting and integrating new components in subsequent trials (T. C. Hoffmann et al., 2013). Poor reporting creates research waste either through inadequate trial replication and extension, or through lost opportunity to implement findings into practice (Glasziou et al., 2014; T. C. Hoffmann et al., 2013). Finally, poor reporting ultimately creates an implementation gap as practitioners and the young people they serve are unable to take timely advantage of potentially effective interventions (Deenik et al., 2019; T. C. Hoffmann et al., 2013).

The method of delivery also needs to better reported, including detailed information regarding who delivered the intervention and their level of training, the environment in which the intervention was delivered, if the interventions were self‐selected based on preference or prescribed, and delivered individually or in groups. Improved reporting of physical activity/exercise interventions could shed light on whether minimum dosages are required to improve mental health outcomes in young people, or whether interventions promoting messaging such as ‘move more, sit less’ and ‘more is probably better’ are sufficient (Teychenne et al., 2020). This will directly benefit both clinical prescriptions of physical activity/exercise and public health guidelines for mental health.

## AUTHOR CONTRIBUTIONS

Michaela Pascoe, Alex Parker, Melinda Craike conducted the literature search; Michaela Pascoe designed the figures and tables; all authors contributed to study design; Michaela Pascoe, Alex Parker, Alan Bailey, Nigel Stepto, Rhiannon Patten, Timothy Carter contributed to data collection; Michaela Pascoe, Alex Parker, Alan Bailey, Nigel Stepto, Rhiannon Patten, Timothy Carter contributed to data analysis and data interpretation; All authors contributed to writing and reviewing the manuscript. [Correction added on 28 September 2020, after first online publication: The author contributions section has been corrected.]

## Data Availability

The data that support the findings of this study are available from the corresponding author upon reasonable request.
